# Supplementary material for: Iterative improvement in the automatic modular design of robot swarms
Source: PeerJ Comput Sci. 2020 Dec 7;6:e322. doi: 10.7717/peerj-cs.322 (PMC7924708; doi:10.7717/peerj-cs.322)
Supplement: Supplemental Information 3 [file peerj-cs-06-322-s003.zip › argos3/doc/api/standalone/a00522_source.html]

ARGoS: plugins/robots/generic/simulator/differential\_steering\_default\_actuator.cpp Source File


- Main Page
- Related Pages
- Namespaces
- Classes
- Files

- File List
- File Members

# plugins/robots/generic/simulator/differential\_steering\_default\_actuator.cpp

Go to the documentation of this file.

```
00001 
00007 #include "differential_steering_default_actuator.h"
00008 #include <argos3/core/utility/logging/argos_log.h>
00009 #include <argos3/core/utility/plugins/factory.h>
00010 
00011 namespace argos {
00012 
00013    /****************************************/
00014    /****************************************/
00015 
00016    CDifferentialSteeringDefaultActuator::CDifferentialSteeringDefaultActuator() :
00017       m_pcWheeledEntity(NULL),
00018       m_pcRNG(NULL),
00019       m_fNoiseStdDeviation(0.0f) {
00020       m_fCurrentVelocity[LEFT_WHEEL] = 0.0f;
00021       m_fCurrentVelocity[RIGHT_WHEEL] = 0.0f;
00022    }
00023    
00024    /****************************************/
00025    /****************************************/
00026    
00027    void CDifferentialSteeringDefaultActuator::SetRobot(CComposableEntity& c_entity) {
00028       try {
00029          m_pcWheeledEntity = &(c_entity.GetComponent<CWheeledEntity>("wheels"));
00030          if(m_pcWheeledEntity->GetNumWheels() != 2) {
00031             THROW_ARGOSEXCEPTION("The differential steering actuator can be associated only to a robot with 2 wheels");
00032          }
00033          m_pcWheeledEntity->Enable();
00034       }
00035       catch(CARGoSException& ex) {
00036          THROW_ARGOSEXCEPTION_NESTED("Error setting differential steering actuator to entity \"" << c_entity.GetId() << "\"", ex);
00037       }
00038    }
00039 
00040    /****************************************/
00041    /****************************************/
00042 
00043    void CDifferentialSteeringDefaultActuator::Init(TConfigurationNode& t_tree) {
00044       try {
00045          CCI_DifferentialSteeringActuator::Init(t_tree);
00046          GetNodeAttributeOrDefault<Real>(t_tree, "noise_std_dev", m_fNoiseStdDeviation, 0.0f);
00047          if(m_fNoiseStdDeviation > 0.0f) {
00048             m_pcRNG = CRandom::CreateRNG("argos");
00049          }
00050       }
00051       catch(CARGoSException& ex) {
00052          THROW_ARGOSEXCEPTION_NESTED("Initialization error in foot-bot steering actuator.", ex);
00053       }
00054    }
00055 
00056    /****************************************/
00057    /****************************************/
00058    
00059    void CDifferentialSteeringDefaultActuator::SetLinearVelocity(Real f_left_velocity,
00060                                                   Real f_right_velocity) {
00061       /* Convert speeds in m/s */
00062       m_fCurrentVelocity[LEFT_WHEEL] = f_left_velocity * 0.01f;
00063       m_fCurrentVelocity[RIGHT_WHEEL] = f_right_velocity * 0.01f;
00064       /* Apply noise */
00065       if(m_fNoiseStdDeviation > 0.0f) {
00066          AddGaussianNoise();
00067       }
00068    }
00069    
00070    /****************************************/
00071    /****************************************/
00072    
00073    void CDifferentialSteeringDefaultActuator::Update() {
00074       m_pcWheeledEntity->SetVelocities(m_fCurrentVelocity);
00075    }
00076 
00077    /****************************************/
00078    /****************************************/
00079    
00080    void CDifferentialSteeringDefaultActuator::Reset() {
00081       m_fCurrentVelocity[LEFT_WHEEL]  = 0.0f;
00082       m_fCurrentVelocity[RIGHT_WHEEL] = 0.0f;
00083    }
00084    
00085    /****************************************/
00086    /****************************************/
00087    
00088    void CDifferentialSteeringDefaultActuator::AddGaussianNoise() {
00089       m_fCurrentVelocity[LEFT_WHEEL]  += m_fCurrentVelocity[LEFT_WHEEL] * m_pcRNG->Gaussian(m_fNoiseStdDeviation);
00090       m_fCurrentVelocity[RIGHT_WHEEL] += m_fCurrentVelocity[RIGHT_WHEEL] * m_pcRNG->Gaussian(m_fNoiseStdDeviation);
00091    }
00092 
00093    /****************************************/
00094    /****************************************/
00095 
00096 }
00097 
00098 REGISTER_ACTUATOR(CDifferentialSteeringDefaultActuator,
00099                   "differential_steering", "default",
00100                   "Carlo Pinciroli [ilpincy@gmail.com]",
00101                   "1.0",
00102                   "The differential steering actuator.",
00103                   "This actuator controls the two wheels a differential steering robot. For a\n"
00104                   "complete description of its usage, refer to the\n"
00105                   "ci_differential_steering_actuator.h file.\n\n"
00106                   "REQUIRED XML CONFIGURATION\n\n"
00107                   "  <controllers>\n"
00108                   "    ...\n"
00109                   "    <my_controller ...>\n"
00110                   "      ...\n"
00111                   "      <actuators>\n"
00112                   "        ...\n"
00113                   "        <differential_steering implementation=\"default\" />\n"
00114                   "        ...\n"
00115                   "      </actuators>\n"
00116                   "      ...\n"
00117                   "    </my_controller>\n"
00118                   "    ...\n"
00119                   "  </controllers>\n\n"
00120                   "OPTIONAL XML CONFIGURATION\n\n"
00121                   "It is possible to specify noisy speed in order to match the characteristics\n"
00122                   "of the real robot. This can be done with the attribute: 'noise_std_dev',\n" 
00123                   "which indicates the standard deviation of a gaussian noise applied to the\n"
00124                   "desired velocity of the steering:\n\n"
00125                   "  <controllers>\n"
00126                   "    ...\n"
00127                   "    <my_controller ...>\n"
00128                   "      ...\n"
00129                   "      <actuators>\n"
00130                   "        ...\n"
00131                   "        <differential_steering implementation=\"default\"\n"
00132                   "                               noise_std_dev=\"1\" />\n"
00133                   "        ...\n"
00134                   "      </actuators>\n"
00135                   "      ...\n"
00136                   "    </my_controller>\n"
00137                   "    ...\n"
00138                   "  </controllers>\n",
00139                   "Usable"
00140    );
00141
```

---

Generated on 10 Jul 2018 for ARGoS by 
 1.6.1
